# TG4010 immunotherapy plus chemotherapy as first-line treatment of advanced non small cell lung cancer (NSCLC): Phase IIb results of the TIME trial

**DOI:** 10.1186/2051-1426-3-S2-P441

**Published:** 2015-11-04

**Authors:** John Nemunaitis, Zsolt Papai, Hervé Léna, Gyorgy Losonczy, Frederic Forget, Christos Chouaid, Alexandra Szczesna, Radj Gervais, Christian H Ottensmeier, Joseph Beck, Andrzej Kazarnowicz, Virginie Westeel, Didier Debieuvre, Anne Madroszyk, Enriqueta Felip, Jean Marc Limacher, Elisabeth Quoix

**Affiliations:** 1 Mary Crowley Medical Research Center, Dallas, TX, USA; 2 Fejer Megyei Szent Gyorgy Korhaz, Szekesfehervar, Hungary; 3 Hôpital Pontchaillou, Rennes, France; 4 Semmelweis Egyetem AOK, Budapest, Hungary; 5 Centre Hospitalier de l'Ardenne, Libramont, Belgium; 6 Centre Hospitalier Intercommunal de Créteil, Créteil, France; 7 Mazowieckie Centrum Leczenia Chorob Pluc i Gruzlicy, Otwock, Poland; 8 Centre François Baclesse, Caen, France; 9 Southampton University Hospitals NHS Trust, Southampton, UK; 10 Highlands Oncology Group, Fayetteville, AZ, USA; 11 SP Zespol Gruzlicy i Chorob Pluc w Olsztynie, Olsztyn, Poland; 12 CHU de Besancon Hopital Jean Minjoz, Besançon, France; 13 CH Mulhouse Hopital Emile Muller Moenchsberg, Mulhouse, France; 14 Institut Paoli Calmettes, Marseille, France; 15 Vall d'Hebron University Hospital, Barcelona, Spain; 16 Transgene S.A., Illkirch, France; 17 Nouvel Hôpital Civil, Strasbourg, France

## Background

TG4010 is an immunotherapy using an attenuated and modified poxvirus (MVA) coding for MUC1 and interleukin-2 to induce a cellular immune response against MUC1 expressing tumors. Previous Phase 2 trials have demonstrated the efficacy and safety of TG4010 in combination with chemotherapy. In addition, level of Triple Positive Activated Lymphocytes (TrPAL; CD16+, CD56+, CD69+) was identified as a potential biomarker predictive of efficacy

## Methods

TIME is a double blind, placebo-controlled Phase IIb/III study. The Phase IIb part compared first-line chemotherapy combined with TG4010 or placebo and further assessed the predictive value of baseline level of TrPAL. Eligibility criteria included previously untreated stage IV NSCLC, MUC1+ tumor by immunohistochemistry, PS ≤1. TG4010 (10^8^ pfu) or placebo was given SC weekly for 6 weeks (w), then every 3w up to progression, in combination with chemotherapy. Primary endpoint was progression-free survival (PFS) and secondary endpoints were response rate (ORR), duration of response, survival (OS), safety and subgroup analyses according to histology and level of TrPAL. (NCT01383148).

## Results

222 pts were randomized 1:1. In pts with normal TrPAL the study met its primary endpoint with a probability >95% that the PFS HR is < 1 in pts treated with TG4010. Preplanned subgroup analyses were performed using an optimal cut-off value for the level of TrPAL defining 2 subpopulations (low and high TrPAL). In 147 patients with low TrPAL, PFS was significantly increased in TG4010 arm (HR=0.66 [CI95% 0.46-0.94] p= 0.010) as well as OS (HR=0.67 [CI95%0.46-0.98) p=0.018) while there was no benefit in pts with high TrPAL. Activity was even more important in patients with low TrPAL and non-squamous tumor (n=127) with PFS HR =0.59 (CI95% 0.40-0.87; p=0.003), and OS HR=0.59 (CI95% 0.39-0.91; p=0.007). In this subgroup, ORR was 39.3% vs 30.3% and duration of response 43.1 vs 18.1weeks in TG4010 and placebo arms, respectively. TG4010 related adverse events were mainly low-grade injection site reactions. The impact of PDL1 expression by immunohistochemistry in the tumor of patients treated with TG4010 supports the activity of TG4010 whether the tumor is positive or negative for PDL1 expression.

## Conclusions

These results provide further evidence of the efficacy of TG4010 in combination with chemotherapy in NSCLC and of the potential of TrPAL as a predictive biomarker. Future development of TG4010 is being planned, both in combination with chemotherapy (Phase 3 part of the TIME trial) and in combination with immune checkpoint inhibitors.

## Trial registration

ClinicalTrials.gov identifier NCT01383148.

